# The miR-106a~363^Xpcl1^ miRNA cluster induces murine T cell lymphoma despite transcriptional activation of the p27^Kip1^ cell cycle inhibitor

**DOI:** 10.18632/oncotarget.16932

**Published:** 2017-04-07

**Authors:** Daniel A. Kuppers, Thomas M. Schmitt, Harry C. Hwang, Lavanya Samraj, Bruce E. Clurman, Matthew L. Fero

**Affiliations:** ^1^ Fred Hutchinson Cancer Research Center, Seattle, Washington, USA; ^2^ Phenopath Laboratories, Seattle, Washington, USA; ^3^ University of Washington, Seattle, Washington, USA; ^4^ University of New Mexico, Albuquerque, New Mexico, USA

**Keywords:** cell cycle, mouse model, T cell development, oncogene, non-coding RNA

## Abstract

The miR-106a~363 cluster encodes 6 miRNAs on the X-chromosome which are abundant in blood cells and overexpressed in a variety of malignancies. The constituent miRNA of miR-106a~363 have functional activities *in vitro* that are predicted to be both oncogenic and tumor suppressive, yet little is known about their physiological functions *in vivo*. Mature miR-106a~363 (*Mirc2*) miRNAs are processed from an intragenic, non-protein encoding gene referred to as Xpcl1 (or *Kis2*), situated at an X-chromosomal locus frequently targeted by retroviruses in murine lymphomas. The oncogenic potential of miR-106a~363*^Xpcl1^* has not been proven, nor its potential role in T cell development. We show that miR106a~363 levels normally drop at the CD4+/CD8+ double positive (DP) stage of thymocyte development. Forced expression of *Xpcl1* at this stage impairs thymocyte maturation and induces T-cell lymphomas. Surprisingly, miR-106a~363*^Xpcl1^* also induces p27 transcription via Foxo3/4 transcription factors. As a haploinsufficient tumor suppressor, elevated p27 is expected to inhibit lymphomagenesis. Consistent with this, concurrent p27*^Kip1^* deletion dramatically accelerated lymphomagenesis, indicating that p27 is rate limiting for tumor development by *Xpcl1*. Whereas down-regulation of miR-106a~363 is important for normal T cell differentiation and for the prevention of lymphomas, eliminating p27 reveals *Xpcl1*'s full oncogenic potential.

## INTRODUCTION

The X-chromosomal miR-106a~363 miRNA cluster is comprised of 6 mature miRNAs which are highly conserved from mice to humans. Constituents of the miR-106a~363 cluster, and its autosomal paralogs (miR-106b~25 and miR-17~92), include some of the most highly expressed miRNAs in blood cells [1]. The component miRNAs of these clusters, are differentially expressed in lymphocyte and megakaryocyte development [2–4]. High levels of miR-106a~363 miRNA have also been observed in a variety of human malignancies. In anaplastic large cell lymphoma (ALCL), an aggressive human T cell lymphoma, miR-106a~363 is highly expressed (especially miR-106a and miR-20b), whereas the same tumor type typically exhibits a PI3K-dependent loss of the Cdk inhibitor protein p27^Kip1^ (Cdkn1b) [5–7]. B cell lymphomas may also express varying levels of miR-106a~363 or its paralogs. In mantle cell lymphoma, a disease characterized by high levels of cyclin D1, elevated levels of miR-20b predicted shortened overall survival [8]. While genetic evidence of the oncogenicity of miR-106a~363 in human malignancies is lacking, amplifications of the miR-17~92 paralog has been documented in diffuse large B cell lymphomas [9].

Despite these associations, the pathophysiological significance of miR-106a~363 is largely unknown. The first indication that miR-106a~363 is potentially oncogenic came with discovery of *Xpcl1* (Kis2), an intergenic non-coding RNA gene that was a recurring target site in several murine retroviral mutagenesis screens [10–12]. *Xpcl1* was targeted at a particularly high frequency in tumors induced in p27^Kip1^ knockout mice (and thus termed the X-linked p27 cooperating locus) [10]. Subsequent work identified the *Xpcl1* gene as a cluster of miRNAs, miR-106a~363, which are cleaved from primary transcripts which span the cluster [13]. Although viral mutagenesis studies have implicated miR-106a~363, they have not specifically proven the oncogenic potential of miR-106a~363 since retroviral LTRs can enhance expression of multiple genes, even at great distances from the viral integration site. Additionally, retroviruses typically integrate at dozens of genomic locations in a single tumor clone, making the relative importance of miR-106a~363 activation unclear. The most direct confirmation of the oncogenic potential of a gene is to induce its expression in a transgenic organism. Still, this approach is not 100% sensitive, since many oncogenes must work in concert with other mutations in order to exhibit a malignant phenotype. Forced expression of the miR-17~92 paralog, for example, required concurrent expression of Myc, to induce B cell lymphomas [14].

In adult tissues, as well as human malignancies, there is significant overlap in the pattern of expression of miR-106a~363 and its paralogs. For example, miR-17~92, miR-106a~363, and miR-106b~25 exhibit sequential and overlapping waves of expression in developing B lymphocytes [15]. Together, the miR-106a~363 miRNAs are predicted to target a hundreds of mRNA transcripts, including multiple cell cycle regulators, and therefore could exhibit either oncogenic or tumor suppressor activity. In glioma cells, for example, miR-106a targets E2F1 and inhibits cell proliferation [16]. On the other hand, miR-106b (which is also a miR-17 family member) targets the p21 Cdk inhibitor (Cdkn1a) and thereby enhances growth of mammary epithelial cells [17]. The importance of Xpcl1 paralogs for normal T-cell growth was indicated in a screen of miRNA that rescue DGCR8-deficient T cells. Multiple miR-17 and miR-92 family members were amongst the miRNA that most effectively induced Th cell proliferation [18].

p27^Kip1^ is a member of the Cip/Kip family of Cdk inhibitor and is capable of inactivating a variety of cyclin/Cdk complexes [19]. Thymocytes from p27^Kip1^ null (p27^−/−^) mice have extraordinarily high levels of Cyclin A/Cdk2 and Cyclin D/Cdk4 catalytic activity [20–22]. In a large M-MuLV mutagenesis screen, increased co-integration of virus at *Xpcl1* and cyclin D3 was noted, suggesting that these two genes cooperate in lymphomagenesis [23]. However, the mechanism of cooperation, if any, between p27 loss and *Xpcl1* activation in lymphomagenesis is unknown. Also, there is no known functional interaction between the miR-106a~363 miRNAs and p27^Kip1^. In the current study we demonstrate that forced expression of miR-106a~363 perturbs normal T cell differentiation, at the DP stage, and induces aggressive T cell lymphomas. We focus on the mechanism of cooperation between p27 loss and *Xpcl1* in lymphomagenesis, and show that p27^Kip1^ deletion circumvents its transcriptional activation by *Xpcl1*.

## RESULTS

### Expression of miR-106a~363 cluster during T cell differentiation

**Figure 1:**
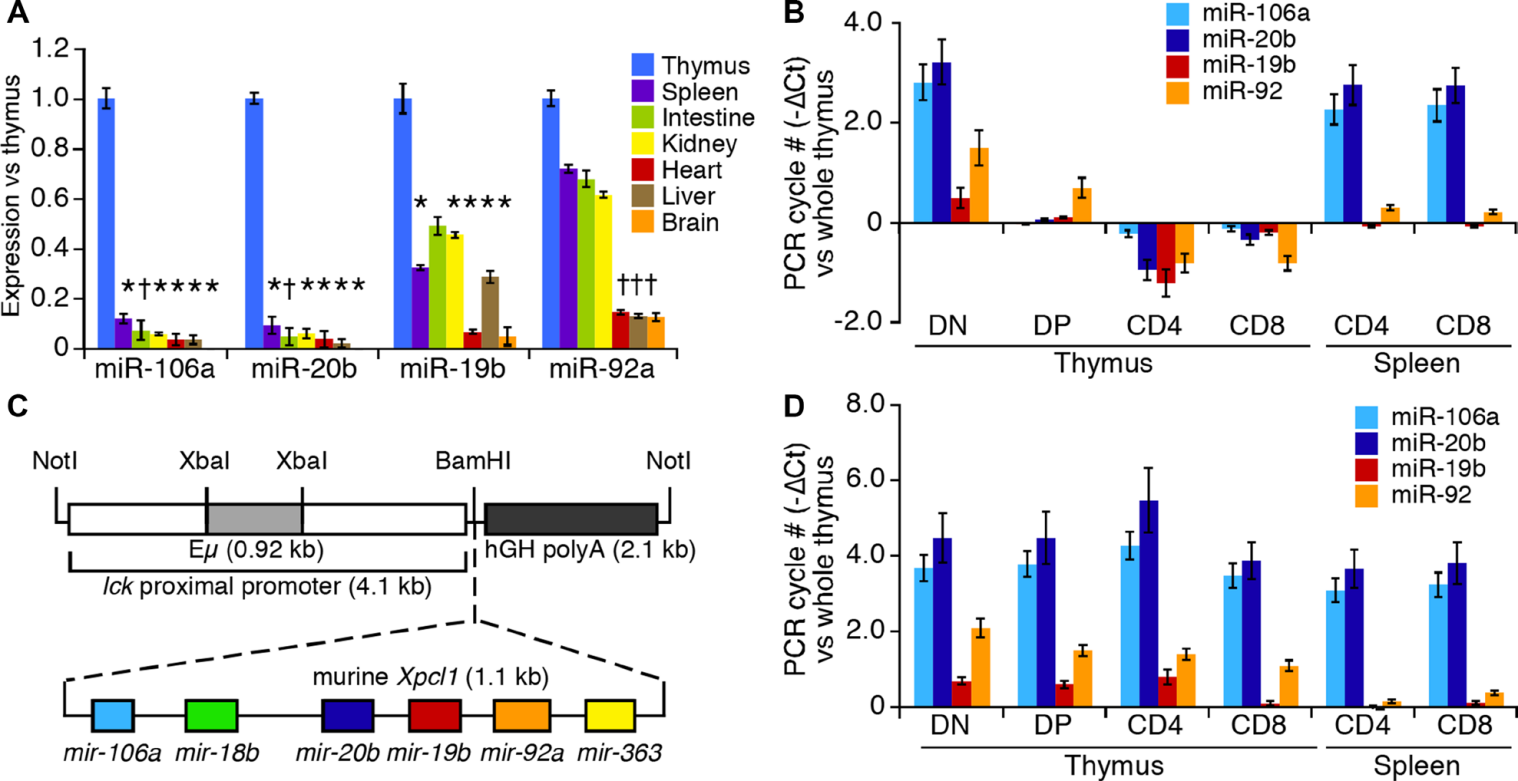
miR-106a~363 expression in wild type and Lx transgenic mice (**A**) Expression of component miRNA from the miR-106a~363 cluster quantified in normal mouse tissues by RT-qPCR. miRNA levels are expressed as fold change relative to whole thymus. (**B**) Levels of the miRNA in T cell subsets from the thymus and spleen relative to whole thymus. (**C**) The Lck-Xpcl*1* (Lx) transgene includes an *Xpcl1* genomic fragment, with the miR-106a~363 cluster, adjacent to the Lck proximal promoter. (**D**) Expression of miR-106a~363 miRNAs in T cell subsets from Lx+ transgenic mice relative to wild type whole thymus. (Values are mean ± s.d., *p < 0.01, ^†^*p* < 0.05, *t-test*).

We compared the expression level of mature miRNAs across different tissues and within T cell subsets by RT-qPCR using primers specific to mature miR-106a~363 miRNA. As shown in Figure 1A, the levels of the four most abundant miRNA from miR-106a~363 are highly expressed in thymus compared to other tissues. Within the thymus (Figure 1B), expression was high in the early stages of differentiation, CD4^–^CD8^–^ double-negative (DN), but dropped significantly in CD4^+^CD8^+^ double positive (DP) thymocytes, and remained low in both CD4^+^ and CD8^+^ single positive (SP) thymocytes. Expression rose again in splenic CD4 and CD8 SP T cells. To study the effect of miR-106a~363 cluster expression in the thymus, we generated transgenic mice by coupling the Lck promoter to a 1.1 kb fragment of murine *Xpcl1* genomic DNA (Figure 1C). The Lck promoter directs T cell specific gene expression at an early stage of thymocyte development. Two independent founder lines exhibited similar expression patterns and cellular phenotype. Notably, the Lck-Xpcl1 (Lx) transgene sustains high-levels of miR-106a~363 miRNA throughout all stages of thymocyte differentiation (Figure 1D), in contrast to wild type (Figure 1B). In contrast, miR-106a~363 expression was unchanged in non-lymphoid tissues of Lx^+^ mice compared to wild type (less than 2-fold changes were seen in skeletal muscle, brain, intestine, heart, kidney or liver by RT-qPCR), which confirms the specificity of the transgene (data not shown).

### Cooperation of Lck-Xpcl1 and p27^Kip1^ loss in T cell lymphomagenesis

**Figure 2:**
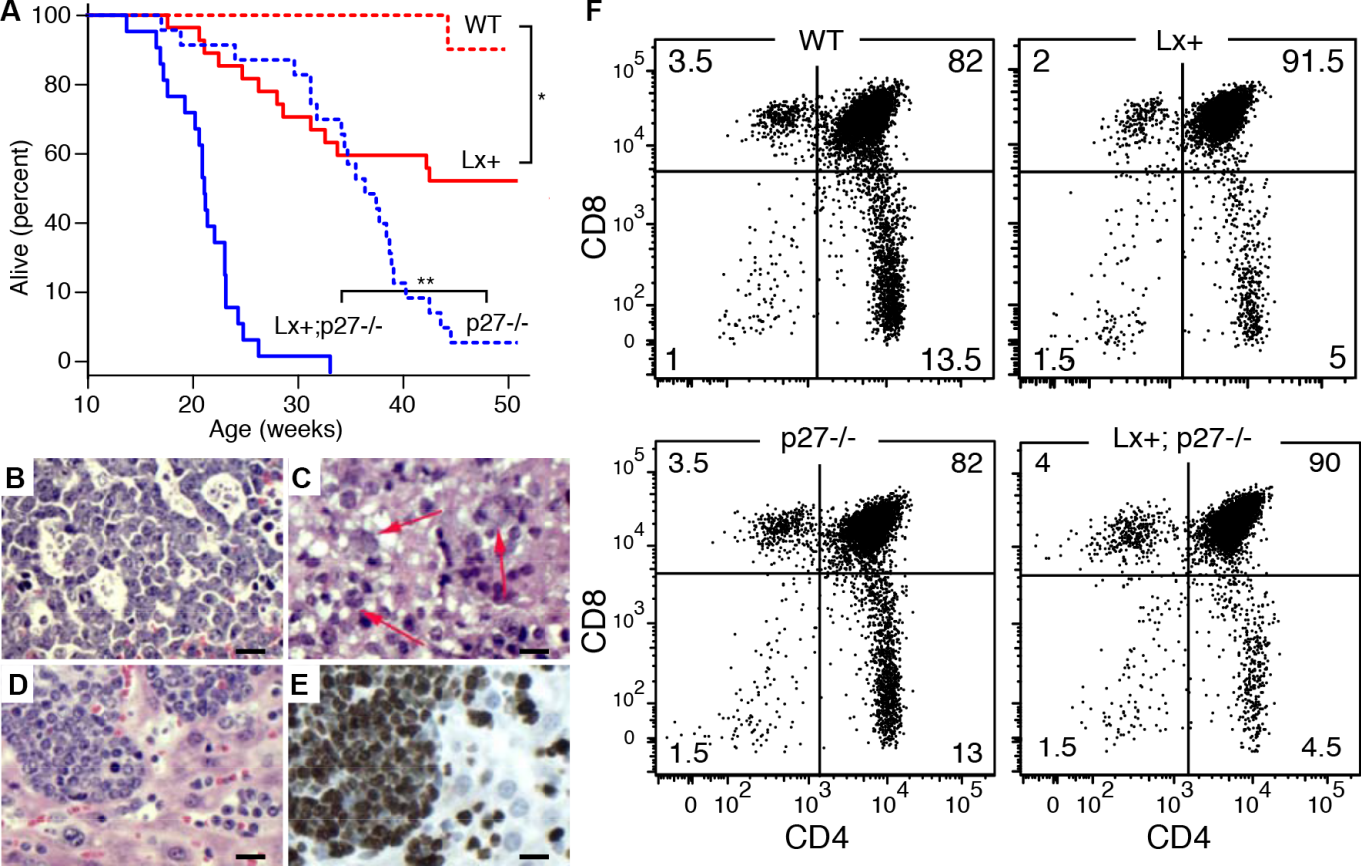
Cooperation with p27^Kip1^ loss in miR-106a~363 induced lymphomas (**A**) Kaplan-Meier survival curves for wild type (WT, *n* = 17), Lx+ (*n* = 17), p27−/− (*n* = 32), and Lx+; p27−/− mice (*n* = 23). (*p = 0.0014, **p < 0.001; Log-rank test). (**B**–**E**) Histological appearances of H&E stained T cell lymphomas. (B) The higher grade tumors often demonstrated blastoid histology (monomorphism with finely dispersed chromatin and easily visualized nucleoli) with scattered epithelioid histiocytes or macrophages imparting a “starry-sky” appearance. (C) A subset of cases contained scattered markedly enlarged and more anaplastic appearing tumor cells containing large clear cytoplasmic vacuoles (arrows), similar to cells seen in human anaplastic large cell lymphoma (ALCL). (D–E) Diffuse lymphomatous tumor infiltrate in the liver is shown with corresponding Ki67 immunostained section showing high proliferation rate, > 95%. (Scale bars = 100 μm) (**F**) Flow cytometry showing CD4 and CD8 cell subsets from 8–10 week old (non-malignant) thymuses in wild type (WT), Lx+, p27^−/−^, and Lx^+^;p27^−/−^ mice. Both Lx^+^ and Lx^+^;p27^−/−^ mice display increased numbers of CD4^+^,CD8^+^ (DP) thymocytes and reduced CD4^+^ SP cells (*p* < 0.001, *t-test*). CD8 subsets are modestly reduced (*p* = 0.04).

Lx^+^ mice exhibit reduced survival at one year (Figure 2A) compared to wild type animals (49% vs. 95%). p27^−/−^ mice also exhibited shortened survival, as expected, due to pars intermedia pituitary tumors [24]. The combination of Lck-Xpcl1 and p27^Kip1^ deletion led to significant reductions in overall survival compared to p27^−/−^ mice. T cell lymphomas were the cause of morbidity in 85% (13/15) of Lx^+^ transgenic mice that became ill by one year of age (Table 1). In contrast, no wild type mice or p27^−/−^ mice developed lymphoid malignancies. Lymphomas were found at a higher frequency in Lx^+^; p27^−/−^ compound mutants. By 25 weeks most of the Lx^+^; p27^−/−^ mice had died from T cell lymphomas. As expected, a subset of these mice had coincidental pituitary tumors, however at this age the pituitary tumors were relatively small. Of the 23 immunophenotyped tumors, most (91%) had a DP immunophenotype and the remainder (9%) were CD4 SP (Supplementary Figure 1A). Tumors frequently caused massive thymic enlargement, but 20% of tumors involved primarily the spleen and lymph nodes. Tumors (from 27 mice) were evaluated using H&E staining plus Ki-67 immunohistochemistry. In general, the tumors had a diffuse growth pattern with intermediate to high grade cytological appearance with numerous mitotic figures (Figure 2B). A subset of cases (*n* = 5) contained scattered markedly enlarged and more anaplastic appearing tumor cells, reminiscent of tumor cells seen in human anaplastic large cell lymphoma (ALCL) (Figure 2C). Ki67 staining was consistently high in Lx+,p27−/− tumors (95 ± 1.6%, mean ± sd) but was more variable in Lx+;p27+/+ tumors (83 ± 19%, *p-value* = 0.048, two-tailed *t* test) (Figure 2D, 2E, Supplementary Figure 1B). The rate of apoptosis was low in both Lx^+^ tumors (caspase-3 cells/mm^2^) and tumors from Lx^+^; p27^−/−^ animals (1.13 ± 0.5 vs. 1.7 ± 0.8 caspase-3 cells/mm^2^, Supplementary Figure 1C). By RT-qPCR, tumor cells exhibited sustained miR-106a~363 levels comparable to pre-malignant Lx^+^ thymocytes (ΔCt = 0 ± 0.3, mean ± sd).

**Table 1:** Cause of morbidity in Lck-Xpcl1 and p27−/− mice

Genotype	*n*	Pituitary tumor	T cell lymphoma	Non-malignant^a^
Wild type	1	0	0	1 (100%)
Lck-Xpcl1^+^	15	0	13 (87%)	2 (12%)
p27−/−	12	11 (92%)	0	1 (8%)
Lck-Xpcl*1*+; p27−/−	18	3 (17%)^b^	16 (89%)	0

a. Non-malignant causes of morbidity included skin lesions, uterine prolapse and gastrointestinal bleeding.

b. 2/3 of Lx^+^; p27^−/−^ mice had concurrent T cell lymphomas.

### Late double-positive (DP) thymocyte developmental block in Lck-Xpcl1 mice

Lx^+^ thymocytes exhibit diminished numbers of CD4 SP cells compared to wild type mice (4.0 vs. 14.7% vs. 4.0%, *n* = 16 mice, *p* < 0.001, *t* test) and CD8 SP cells (3.9% vs 4.8%, *p* = 0.038), with a commensurate increase in the number of DP thymocytes (90.9% vs. 79.2%, *p* < 0.001) (Figure 2F). In contrast, p27^−/−^ mice exhibited no change in the proportion of CD4/CD8 subsets, despite increased overall cellularity, consistent with prior reports [20, 25, 26]. Furthermore, Lx^+^;p27^−/−^ mice exhibited a combined phenotype equivalent to the T cell subset distribution of Lx^+^ mice, plus the hypercellularity seen in p27^−/−^ thymuses.

Several pathophysiologic mechanisms could account the altered T cell subset distributions in Lx^+^ thymocytes. We ruled out a defect in TCRα chain rearrangement (by qPCR quantification of the frequency of Vα-Jα joining) as well as accelerated emigration of SP cells or reduced apoptosis (Supplementary Figure 2A). However, Lx^+^ mice exhibited reduced cell expression of the TCR complex (TCRβ and CD3), and CD69, suggesting impairment in positive selection and progression from the DP stage (Supplementary Figure 2B–2D) [27]. Notably, a large fraction of Lx^+^ CD4 SP and CD8 SP thymocytes express low levels of CD24 comparable to that of peripheral T cells. Thus most of the CD4 and CD8 SP cells in the Lx^+^ thymus have the appearance of recirculating peripheral T cells, pointing to a more profound block to differentiation than indicated by the CD4/CD8 profile alone (Supplementary Figure 2E) [28].

To confirm the late DP stage maturation defect, we assayed thymocyte maturation kinetics in Lx^+^ transgenic mice with two different BrdU labeling schedules: a brief pulse-label of BrdU to measure proliferation and a continuous administration to measure the stage specific maturation time, as previously described [29]. We detected a reduction in Lx^+^ thymocyte proliferation, measured as a reduction in cells in S-phase (1.3 ± 0.2%, mean ± s.d.) compared to wild type animals (2.4 ± 0.5%, Supplementary Figure 3A). With continuous BrdU labeling, an increased number of days was required for BrdU labeled thymocytes to fill the DP compartment of Lx+ mouse thymuses (Supplementary Figure 3B). There was, however, no change in the rate of apoptosis of of Lx+ thymocytes in response to dexamethasone (Supplementary Figure 3C–3D). In summary, miR-106a~363 overexpression results in a shift in thymocyte subset proportions due to slowed progression through the DP stage, as opposed to accelerated emigration of SP cells or reduced apoptosis.

### Regulation of p27^Kip1^ by miR-106a~363

**Figure 3:**
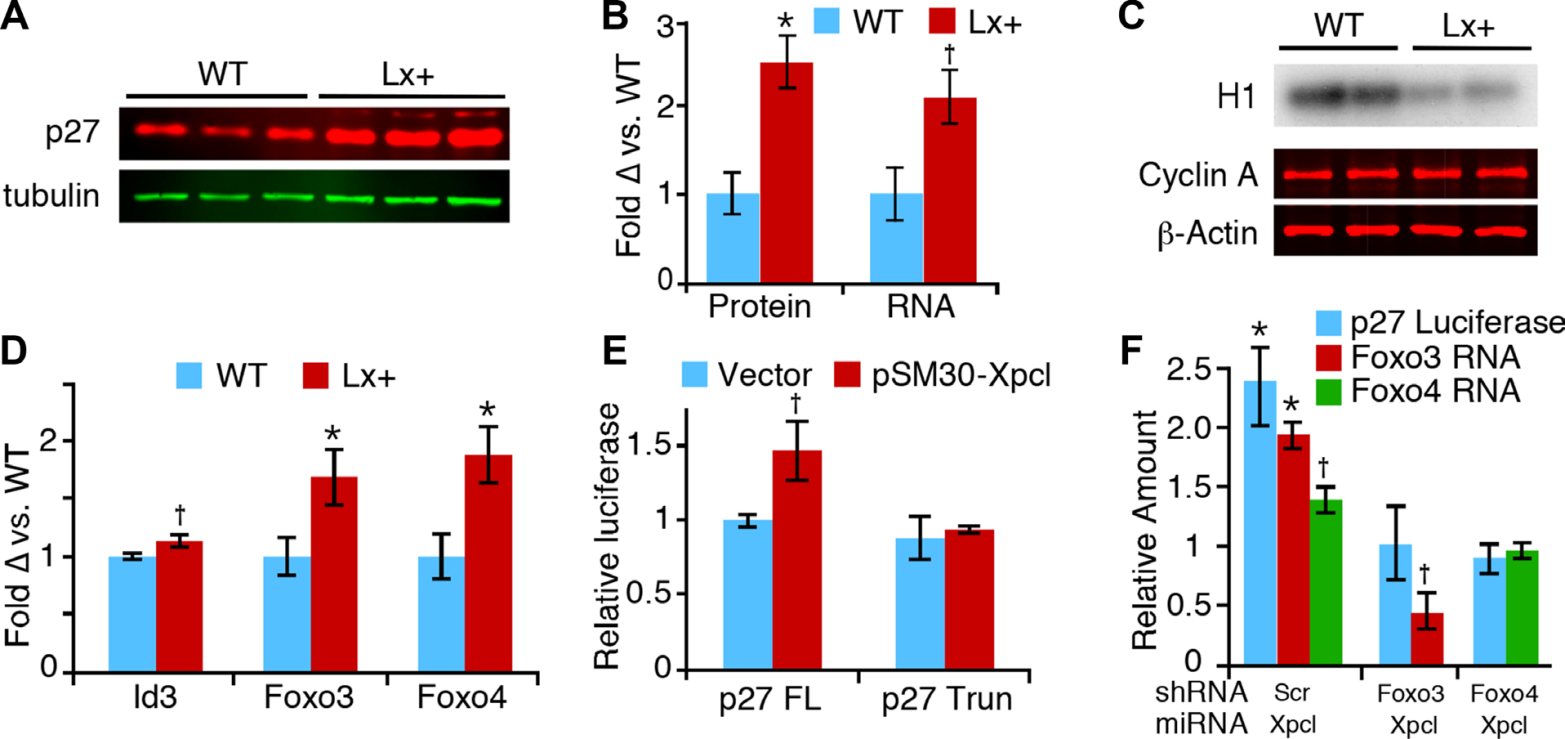
Regulation of p27^Kip1^ expression by miR-106a~363 (**A**) Western blot for p27, and tubulin control, from wild type (WT) and Lx+ thymuses. (**B**) Odyssey image quantitation of p27 protein expression (left), and p27 RNA expression (right), demonstrates comparable elevation of p27 protein and RNA (mean ± SEM). (**C**) Cyclin A-associated histone kinase activity (H1) is reduced in Lx+ thymus, despite unchanged cyclin A protein levels. (**D**) Levels of Foxo3 and Foxo4 mRNA in Lx+ thymus relative to wild type, measured by RT-qPCR. (**E**) *Xpcl1* expression (pSM30-*Xpcl1*) increased luciferase activity in T cells transfected with full length p27-promoter reporter (p27FL) but not the activity of the truncated promoter (p27 Trun). (**F**) Cotransfection of shRNA targeting Foxo3 and Foxo4 block the induction of full length p27 promoter reporter activity (blue bars) by pSM30-Xpcl1 (Xpcl) relative to scrambled shRNA (Scr). Concurrent measurement of Foxo3 and Foxo4 RNA levels by RT-qPCR (green and red bars) show corresponding reductions by the shRNA. (*p < 0.01, ^†^*p* < 0.05, *t-test*).

Considering the synergistic effects of miR-106a~363 and p27^Kip1^ on tumor development, it is plausible that the miRNA cluster cooperates with p27 loss by reducing expression of other cell cycle inhibitors or by secondary cell cycle gene activation. We assessed RNA expression levels of multiple cell cycle genes, in normal 8-week old Lx^+^ transgenic thymus (Supplementary Figure 4A). With the exception of p57, which was increased in Lx^+^ thymus, we found no significant changes in their steady state expression of other cell gene expression, despite the fact that many are predicted targets of miR-106a~363. Surprisingly, we found that p27^Kip1^ was increased 2-3 fold in Lx^+^ thymocytes, at both the RNA and protein level (Figure 3A, 3B). Consistent with the increase in p27^Kip1^, Lx+ thymus also exhibits reduced cyclin A-associated kinase activity (Figure 3C). We assayed levels of mature miRNAs (miR-181a, miR-221 and miR-222) and the Id3 transcription factor, which have been shown to repress p27^Kip1^, but found no changes that would account for increases in p27 (Supplementary Figure 4B, Figure 3D). In contrast RNA levels of Foxo3 and Foxo4, transcription factors which regulate p27 expression, were both increased, and miR-221, which represses p27 expression, was increased [30–33]. (Figure 3D). We confirmed these results *in vitro* by overexpressing miR-106a~363 in two different murine T-cell lines. Human p27 mRNA was also induced by overexpressing the miRNAs in 293T cells, demonstrating that the effect on p27 is not mouse cell specific (Supplementary Figure 4C, 4D). In a luciferase reporter assay, miR-106a~363 increased activity of the p27^Kip1^ promoter, providing further evidence that the effect of miR-106a~363 on p27 is mediated at the transcriptional level. A truncation mutant of the p27 promoter, which removes the Foxo binding site as previously described, eliminated the ability of miR-106a~363 to increase p27 expression (Figure 3E) [32]. We observed reduced responsiveness of the full length p27 promoter to miR-106a~363 when either Foxo3 or Foxo4 expression was knocked-down by shRNA (Figure 3F). From these data we conclude that miR-106a~363 transcriptionally activates p27^Kip1^ in thymocytes via increased Foxo transcription factor expression, ultimately resulting in inhibition of Cdk catalytic activity.

### Regulation of p27^Kip1^ in developing T cells and lymphomas

**Figure 4:**
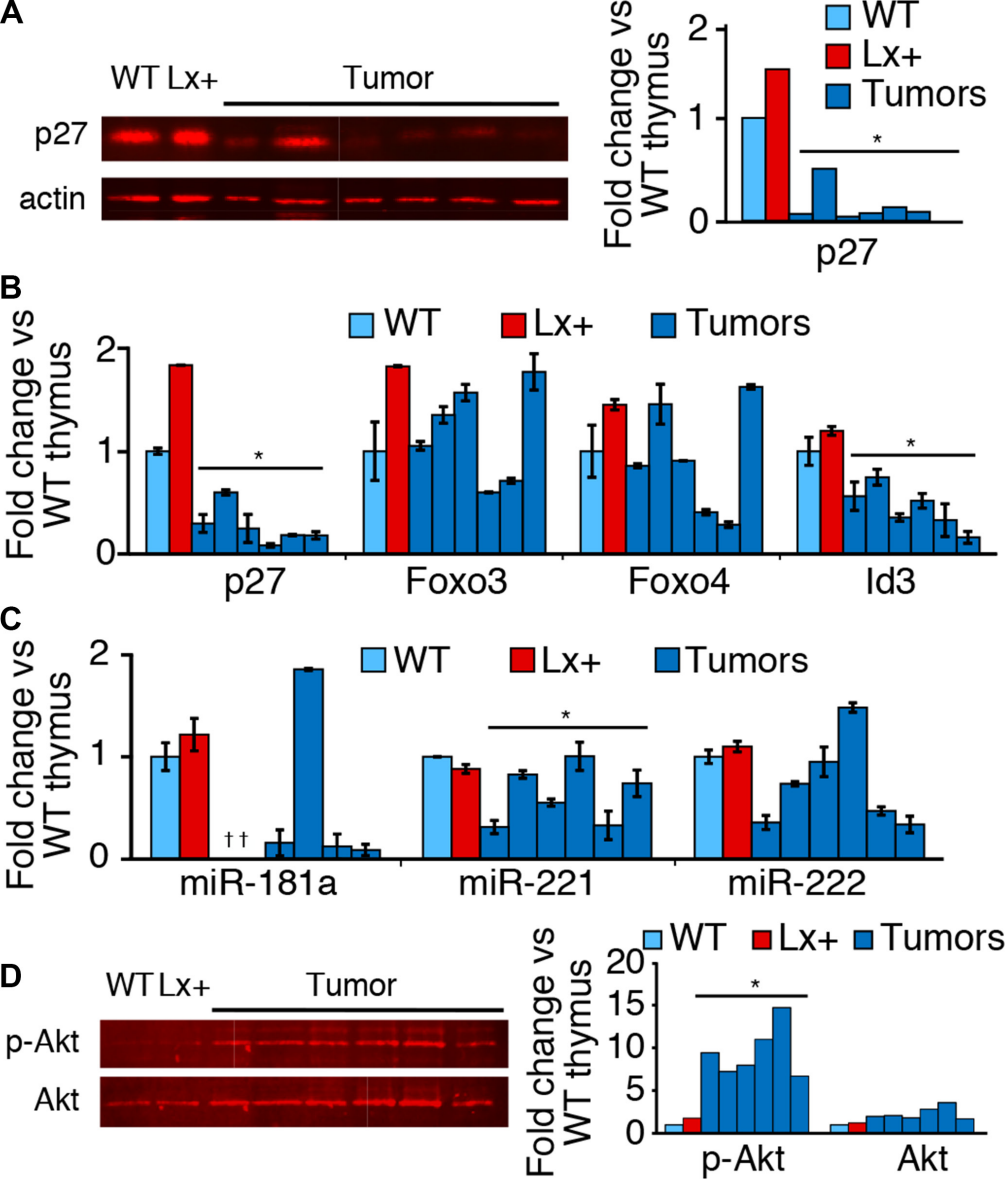
p27 and Foxo expression in tumors and T cell subsets (**A**) Western blot for p27 protein expression from Lx+ thymic lymphomas, wild type thymus (WT) and (Lx+) transgenic mice thymus demonstrates reduced p27 in tumors. Western blots quantified p27 by fluorescent imaging. (**B**) RT-qPCR quantitation of p27, Foxo3, Foxo4, and Id3 RNA from the Lx^+^ tumors, expressed relative to wild type (WT) thymus. p27 and Id3 are reduced in Lx^+^ tumors, compared to wild type and Lx^+^ thymus, whereas FoxO transcription factors are variable. (**C**) Levels of miRNAs, miR-181a, miR-221, and miR-222 (which target p27^Kip1^) quantified by RT-qPCR from Lx+ tumors and normal thymus (*not detected). (**D**) Western blot demonstrating increased phosphorylated Akt (Ser473) in Lx+ lymphomas compared to wild type (WT) and Lx+ thymus. Total Akt levels were relatively unchanged. (Right) quantitation of phospho-Akt and total Akt westerns. (All values are mean ± s.d., *p < 0.05, *t-test*).

We posited that increased p27 and oncogenesis by *Xpcl1* represent two independent activities and that, in the presence of an intact p27^Kip1^ gene, *Xpcl1*-induced tumors must first overcome the increased p27. Consistent with this hypothesis, p27 expression is reduced in Lx^+^ tumors compared to Lx^+^ or wild type thymus (Figure 4A). To determine if p27 down-regulation in tumors was due to transcriptional or post-transcriptional regulation, we measured p27 RNA levels, as well the transcription factors and miRNAs that regulate p27 expression. In addition to reduced p27 protein, Lx^+^ tumors also expressed p27 RNA below that of normal thymus (Figure 4B). However, a comparable drop in Foxo3 and Foxo4 RNA expression was not observed. Neither was reduced p27 RNA associated with increases in any of three miRNA which reportedly target p27^Kip1^ (Figure 4C). Therefore, reduced tumor p27 expression is not due to a reversal of the effect of miR-106a~363 on Foxo3 and Foxo4 transcription. Still, loss of Foxo3 and Foxo4 at the protein level may account for loss of p27 expression, since phosphorylation and destabilization is the primary mode of regulation for these factors. Endogenous thymic Foxo levels are normally undetectable by western blot, but we observed elevated levels of phosphorylated Akt (the primary post-transcriptional regulator of Foxo3/4) in Lx^+^ tumors compared to normal thymus (Figure 4D).

Since miR-106a~363 is differentially regulated in developing thymocytes, and since the regulation of p27 during thymocyte differentiation is not well characterized, we assessed the expression of both p27 and its regulatory factors in T-cell subsets. We found differential expression of p27^Kip1^ at both the protein and RNA levels, with lowest levels in DN cells and the highest levels in DP cells (Supplementary Figure 4E). Furthermore, the RNA expression of Foxo3 and Foxo4 both mirrored that of p27, while Id3 expression, a transcriptional repressor of p27^Kip1^, did not change substantially (Supplementary Figure 4F). These data are consistent with the hypothesis that p27^Kip1^ is transcriptionally up-regulated by Foxo transcription factors at the DP stage of thymopoiesis. Lx^+^ mice, which sustain high miR-106a~363 levels, achieve supernormal levels of p27 in thymocytes.

## DISCUSSION

Although the miR-106a~363*^Xpcl1^* locus is a frequent viral integration site in retrovirus induced lymphomas, little has been previously reported about the role of miR-106a~363, or its paralogs, in lymphomagenesis and normal development in T cells. In this study we show that miR-106a~363 is down regulated during thymocyte differentiation and that this impacts normal T cell development and prevents the development of lymphomas. Other than retroviral mutagenesis studies, which typically induce dozens of mutations, the Lx transgenic mouse is the first example of T cell lymphomas induced by aberrant expression of non-coding RNA. We further show that p27 transcription is normally activated during thymocyte differentiation, that transcription of p27 is enhanced by miR-106a~363, and that p27 is rate limiting to tumor development.

We observed high levels of miR-106a~363 expression in immature DN thymocytes, followed by a drop in expression following the transition to the DP stage. These results are consistent with previously published miRNA sequence data, which indicated that expression of miR-17 family members, as a group, are high in DN thymocytes [2]. Forced expression of miR-106a~363 with the Lck-Xpcl1 transgene led to the development of high-grade T cell lymphomas. The tumors monomorphic histologic appearance and the uniform immunophenotype are consistent with a clonal neoplasm, and confirms the hypothesis that dysregulated *Xpcl1* is oncogenic. We were surprised to find that forced expression of miR-106a~363, alone, induced lymphomas, considering that overexpression of the paralogous cluster, miR-17~92, caused lymphoid hyperplasia, but not spontaneous lymphomas [14, 34]. Whereas the oncogenic potential of miR-17~92, was dependent on miR-19a and associated with impaired apoptosis, the Lx^+^ transgene induced only minimal increases in miR-19b, with no appreciable reduction in apoptosis in Lx^+^ thymocytes. Thus the oncogenic mechanism appears to be fundamentally different in Lx^+^ mice than was previously reported for miR-17~92. Considering the compensatory activities of the paralogous miRNA clusters, it is possible that the mechanism of oncogenesis is not cluster-specific, but instead varies by cell type, and that forced expression of miR-17~92 would be equally oncogenic in T cells.

The altered immunophenotype of Lx^+^ thymocytes may provide clues to the oncogenic mechanism of *Xpcl1* and its role in normal T cell development. Reduced TCR expression in Lx^+^ thymocytes may cause an impairment to positive selection, resulting in the accumulation of DP thymocytes [35]. Mutations that cause a block to cellular differentiation may cooperate with mutations that activate cell proliferation in human myelodysplasia and acute leukemias [36]. Similarly, in mice, both ATM deletion and activating mutations of β−catenin have been shown to induce a DP-stage maturation block, and T-cell lymphomas [37, 38]. Although we did not explore the pattern of expression of Xpcl1 in mature peripheral T-cell subsets, it has been reported that Th17 thymocytes have a lower level of miR-20b expression and that ectopic expression of miR-20b impairs Th17 differentiation, and STAT3 as a potential target [39].

Surprisingly, expression of *Xpcl1* increases p27^Kip1^, despite its overall oncogenic effect. This is seemingly paradoxical, because p27 is a well-established tumor suppressor [40, 41]. Considering that tumor suppression by p27 is gene dose-dependent, overcoming the increased p27 may be a universal requirement in *Xpcl1*-associated lymphomas [40, 42, 43]. Consistent with this idea, we observed low levels of p27 expression in Lx^+^ tumors from mice with an intact p27^Kip1^ gene. The dramatically shortened tumor latency in Lx^+^;p27^−/−^ animals confirms the hypothesis that *Xpcl1* cooperates with p27 loss in tumorigenesis and indicates that p27 is rate limiting to tumor development by miR-106a~363. The fact that Lx^+^;p27^−/−^ mice display an accumulation of DP thymocytes, equivalent to that of Lx^+^; p27^+/+^ mice indicates that p27 does not mediate the T cell maturation defect of Lx^+^ thymocytes, rather it reduces the subsequent progression to high grade lymphoma.

That the activation of p27 by miR-106a~363 occurred at the transcriptional level, was also unexpected. Although the increase in Foxo3 and Foxo4 may completely explain the increased p27 transcription in Lx^+^ thymocytes, it is notable that these transcription factors themselves are also regulated by miR-106a~363 at the RNA level. Whereas the inhibition of Foxo3 and Foxo4 by post-transcriptional modification is well established, their transcriptional regulation is less well understood [44]. In contrast to normal thymus, Lx^+^ lymphomas exhibited *reduced* p27 RNA expression, without corresponding changes in miR-106a~363, Foxo3 or Foxo4 RNA. However, the lymphomas exhibited elevated levels of phospho-Akt, which has been shown to reduce p27 expression both by transcriptional and post-transcriptional mechanisms [45–48]. In conjunction with Raf, Akt activation reduces p27 RNA expression via post-translational modification of the Foxo transcription factors [49]. Akt is also one of several kinases capable of directly phosphorylating and destabilizing p27 [46]. Our data indicate that, in lymphomas, the effect of miR-106a~363 on p27 expression is overridden by the activation of upstream signaling pathways, including Akt. Consistent with the role of the Akt pathway in miR-106a~363 lymphomagenesis, the *Xpcl1* locus was frequently co-mutated with PIK3R5 in a prior retroviral mutagenesis screen [23].

Perhaps the best human correlate with this mouse model is anaplastic large cell lymphoma (ALCL), a T-cell lymphoma characterized by activating mutations of anaplastic large cell kinase (ALK). ALK mutations lead to PI3K/AKT activation, inhibition of Foxo3 through phosphorylation and cytoplasmic localization, and loss of p27 expression [5, 6, 50–52]. We observed a subset of tumors in Lx^+^ mice that contained cells reminiscent of ALCL hallmark cells, that occurred independent of p27 genotype. Interestingly, overexpression of miRNAs from miR-106a~363 and the paralogous cluster, miR-17~92, are both highly characteristic of ALK-positive ALCL [7]. Furthermore, ALK may play a direct role in Xpcl1 paralog activation, as knockdown of ALK led to reduced expression of miRNA from both the miR-17~92 and miR-106a~363 clusters [53]. Xpcl1 paralog overexpression does not appear to be a general characteristic of T-cell lymphomas, to the contrary, PTCL/NOS showed significantly reduced expression of miR-17~92 and miR-106a~363 miRNAs compared to normal T-lymphocytes [54].

It is not unprecedented that *Xpcl1*, as an oncogene, should also display a mixture of anti-oncogenic effects. For example, forced expression of Myc induces increased apoptosis in normal B-lymphocytes, whereas co-expression of Bcl2 enhances Myc-induced lymphomagenesis [55]. Other genes that exhibit mixed or context-dependent oncogenic and anti-oncogenic effects include E2F1, K-Ras, TGFβ and Klf4.[56–60] Klf4 overexpression, for example, induces a p21-dependent cell cycle arrest, but also enhances oncogenesis in conjunction with Ras. Perhaps then, it should not be surprising that the miR-106a~363 miRNAs, which together have hundreds of predicted targets, induces a combination of oncogenic and anti-oncogenic effects. It remains unknown whether the T cell lymphomas that arise in Lx^+^; p27^−/−^ mice remain sensitive to p27 after the initial transformation events. Likewise, it remains to be seen, whether inhibition of miR-106a~363*^Xpcl1^* in established tumors can block their growth or aggressive behavior, thereby signifying potential as a therapeutic target.

## MATERIALS AND METHODS

### Quantitation of miRNA and mRNA

Total RNA was isolated with Trizol (Invitrogen) according to manufacturers instructions, with 0.75 v/v of isopropanol, or by miRNeasy mini kit (Qiagen). Quantification of miRNA was performed using a stem-loop RT primer, followed by qPCR, as previously described (primers listed in Supplementary Table 1) [61]. The specificity and linearity of each miRNA assay was confirmed with oligonucleotide standards. (Supplementary Table 3) mRNA was quantified by oligo-dT RT, followed by qPCR (primers listed in Supplementary Table 2).

### Lx^+^ transgenic and Lx^+^; p27^−/−^ compound mutant mice

A 6 kb Not I fragment containing the Lck promoter and murine genomic *Xpcl1* was excised from pLck-Xpcl (previously described) and injected into C57B6JxCBA F2 hybrid zygotes, to create Lx transgenic mice [62]. Two founder mice, which expressed increased miR-106a~363 miRNAs in T cells, were maintained as independent lines through 129S4 strain backcrosses, and genotyped by PCR with transgene specific primers. Lx^+^ mice were backcrossed to p27^Kip1^ knockouts (carrying the p27L- mutation, herein referred to as p27-) for three generations to produce p27^−/−^ mice, Lx^+^ mice, and Lx^+^; p27^−/−^ compound mutants as littermates [22]. Cohorts of 17-32 mice were observed until one year of age or until mice met predetermined morbidity criteria. The gross cause of morbidity was determined at necropsy, with histologic assessment of all tumor masses or enlarged organs. Kaplan-Meyer survival curves were generated using R software's (http://r-project.org) *survival* package, with *P*-values based on a log-rank test. Mouse studies were approved by the FHCRC IACUC.

### Histology and flow cytometry

Tissue specimens, fixed in 10% NBF and paraffin embedded, were serially cut in 4 μm sections for H&E staining and immunohistochemistry. Tumors were scored with regards to cell size, grade, and proliferation rate by an ABP-certified pathologist, blinded to genotype. Ki-67 immunostaining (clone SP6, ThermoScientific), as previously described [63]. To assay apoptosis, sections were also immunostained with anti-cleaved Caspase-3 (Biocare Medical, CP229B). IHC for CD4 and CD8 was performed on 5 μm frozen sections with rat biotinylated antibodies (BD Biosciences), as above. Matched isotype controls were run for each sample. Thymocytes from 6 to 10 week old mice were suspended in PBS and incubated with anti-mouse CD3 (clone 17A2), CD4 (GK1.5), CD8 (53–6.7), TCRß (H57-597), or CD69 (H1.2F3) (all BD Biosciences). Flow cytometry and sorting was performed on Canto2 and FACSAria machines (BD Biosciences). Data was plotted with bi-exponential scaling using FlowJo software (TreeStar, Inc.) *In vivo* BrdU labeling kinetics were assessed following a single BrdU injection, and following continuous (twice-daily) BrdU injections, as previously described [29].

### Cell culture and luciferase reporter assays

SV40-180 and LGY-6871 murine T cells lines were grown in complete RPMI (media plus 10% v/v FBS, 100 μM β-mercaptoethanol, 50 U/ml penicillin and 50 μg/ml streptomycin) at 37°C and 5% CO_2_. Both cell lines were transfected by electroporation using 10 million cells/mL in 100 μl RPMI with 10% FBS, 50 mM trehalose and up to 20 μg DNA in a 0.2 cm cuvettes at 150V, 975 μF, or in a volume of 250 μL at 270V, 975 μF. The apoptosis assays were performed as previously described [64]. The 1.1 kB *Xpcl1* mouse genomic DNA fragment, from pLck-Xpcl, was cloned into the pSM30 miRNA expression vector [62]. A p27^Kip1^ promoter-luciferase reporter construct (p27Luc) has been previously described [65]. To quantify the effect of miR-106a~363 miRNAs and FoxO transcription factors on p27^Kip1^ expression, p27Luc was electroporated into SV40-180 murine T cells, with pSM30-Xpcl vs. pSM30-empty, plus FoxO shRNA vector vs. scramble control (Open Biosystems, V3LHS_375386, V3LHS_358494), and a Renilla transfection control (pRL-TK, Promega).

### Quantitative western blots and kinase assays

Protein extracts of whole thymus, and flow sorted cells from normal 8-week old mice were prepared as previously described [22]. Western blots for p27^Kip1^ were performed with anti-mouse p27 antibody (described by Chien *et al*), anti-Akt or phospho-Akt-Ser473 (cat# 4060, 9272 Cell Signaling Technologies), with anti-ß-tubulin (T-0198, Sigma) used as loading control. Fluorescently labeled secondary antibodies goat anti-rabbit IgG-IRDye800 (Rockland), or goat anti-mouse IgG Alexa Fluor 680 (BD-Molecular Probes), were quantified on an Odyssey Imager (Li-Cor Biosciences). p27 assay linearity was confirmed with standard curves using admixtures of p27^+/+^ and p27^−/−^ thymic extracts. Monochromatic fluorescent images are displayed individually or as superimposed false-colored images representing detector wavelengths (800 nm red, 680 nm green). Cyclin-A/Cdk2 catalytic activity was assayed as previously described, using 200 μg/100 μL whole thymus extracts in kinase buffer, with rabbit anti-cyclin A antibody (Santa Cruz) [66]. Images were quantified with Phosphorimager autoradiography (G.E.) and ImageQuant software.

## SUPPLEMENTARY MATERIALS FIGURES AND TABLES


