# Supplementary material for: The miR-106a~363Xpcl1 miRNA cluster induces murine T cell lymphoma despite transcriptional activation of the p27Kip1 cell cycle inhibitor
Source: Oncotarget. 2017 Apr 7;8(31):50680–91. doi: 10.18632/oncotarget.16932 (PMC5584189; doi:10.18632/oncotarget.16932)
Supplement: Supplementary file 1 [file oncotarget-08-50680-s001.pdf]

The miR-106a~363^{Xpcl1} miRNA cluster induces murine T cell lymphoma despite transcriptional activation of the p27^{Kip1} cell cycle inhibitor

Supplementary Materials

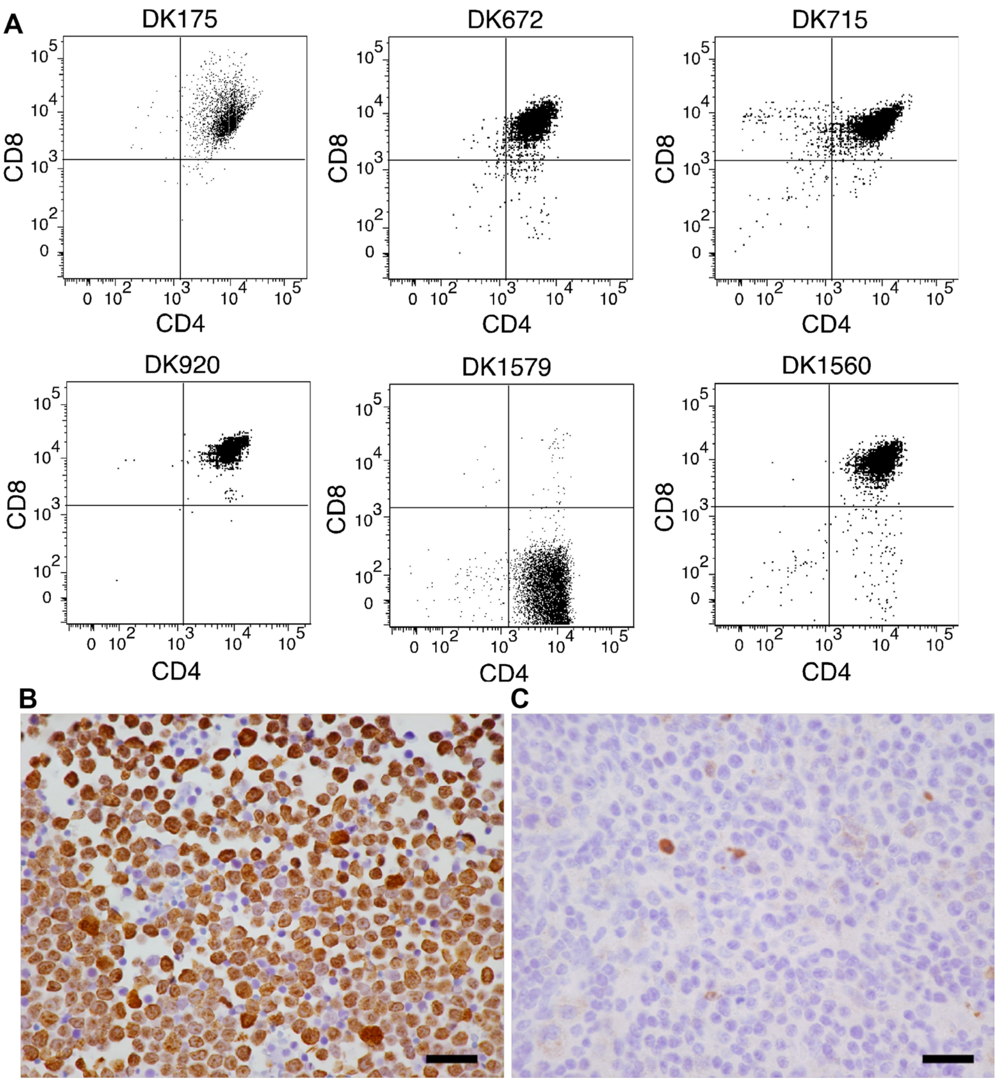

Supplementary Figure 1: (**A**) Flow cytometry analysis of CD4 and CD8 cell surface antigens from Lx+ thymic lymphomas in 6 mice. The tumors samples were prepared and stained using the same method described for whole thymus. Thymic lymphoma showing immunohistochemical staining for Ki67 (**B**) and Caspase-3 (**C**) in consecutive 0.4 µm FFPE sections (hematoxylin counterstain) Scale bar = 100 µm.

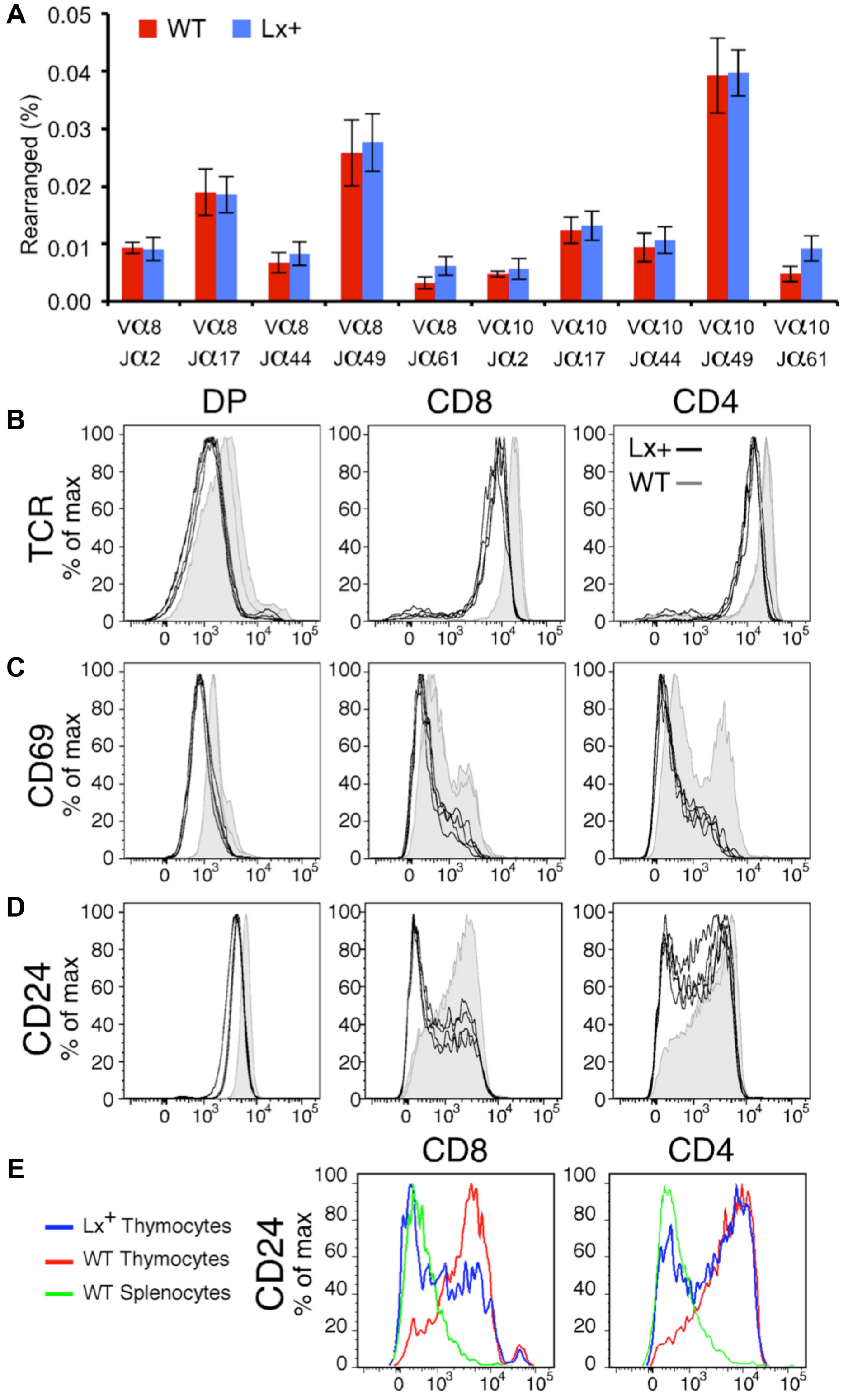

Supplementary Figure 2: (**A**) The frequency of specific TCR Vα-Jα gene rearrangements in flow sorted DP thymocytes from Lx+ mice and wildtype (WT) littermates, quantified by qPCR and normalized to expression of TCR Cα. QPCR was performed and TCRα V-J gene rearrangements was calculated on genomic DNA purified from CD4/CD8 DP thymocytes, using Power SYBR-Green PCR Master Mix (ABI) in a ABI 7900 Real-Time PCR System.(Vacchio et al, 2007) (**B**–**D**) Flow cytometry histograms of thymocyte DP, CD4 SP and CD8 SP subsets immunostained for the T cell receptor (TCR), and for the CD69, and CD24 cell surface markers, with curves for Lck-Xpcl transgenic mice (Lx+) over-laid on wildtype profiles (WT). Surface TCR levels are reduced in Lx+ T cells at all stages. CD69, a marker of positive selection, is reduced in DP cells. CD24, a marker of immature T cells, is absent on the majority of CD4 SP and CD8 SP cells. (**E**) Comparison of CD24 surface expression on CD4 SP and CD8 SP wildtype thymocytes, Lx+ thymocytes and wildtype splenocytes. The expression level of CD24 in the low population from Lx+ thymocytes overlaps with the wildtype splenocyte expression profile, consistent with the population consisting of recirculating peripheral T cells.

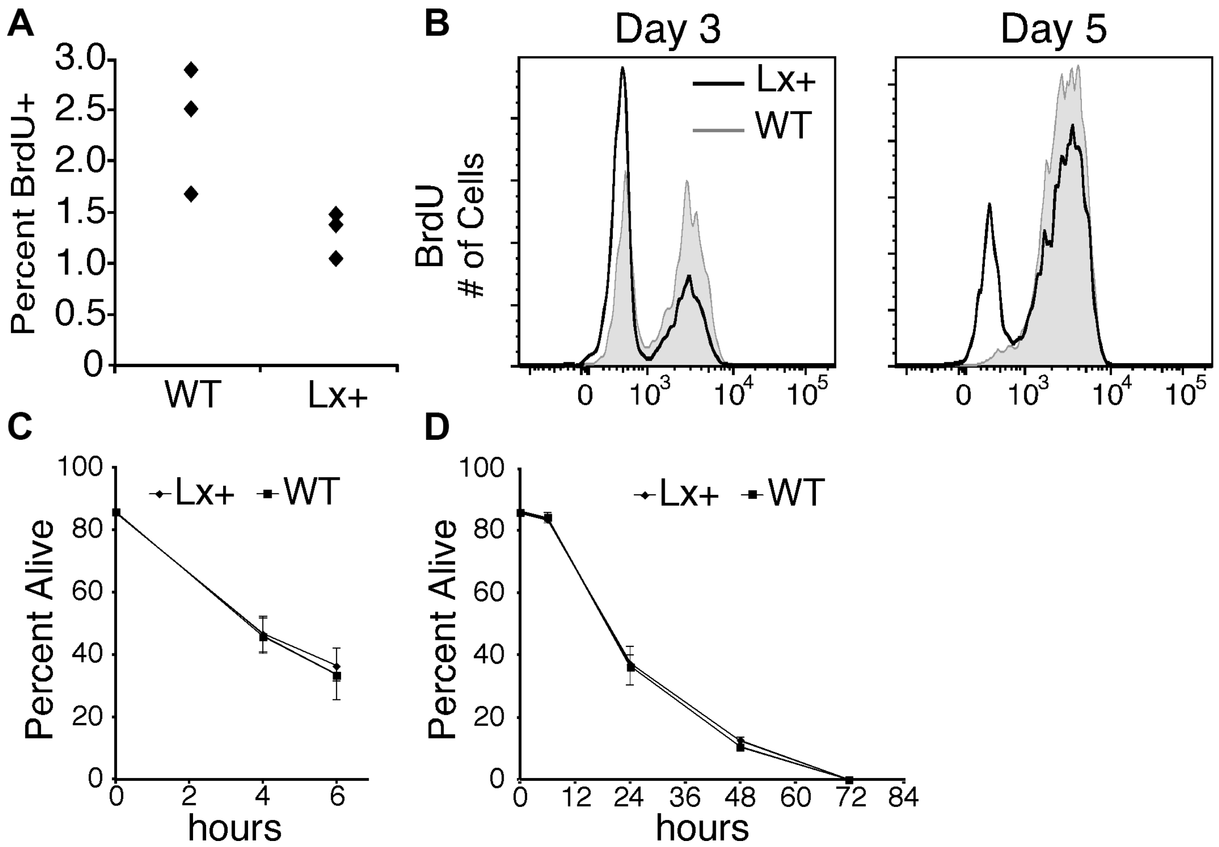

Supplementary Figure 3: Thymocyte proliferation, maturation kinetics and apoptosis. (**A**) Percentage of thymocytes immunostaining positive for BrdU from wildtype (WT) and Lx$^+$ transgenic mice, pulse labeled with BrdU *in vivo* (n = 3 mice per genotype). (**B**) BrdU immunostaining of DP T cells, assessed by flow cytometry, from Lx$^+$ transgenics and wildtype mice (WT), following 3 or 5 days of twice daily BrdU administration (30 mg/kg/dose). A delay in accumulation of BrdU+ cells in the DP compartment is observed in Lx$^+$ mice compared to wildtype controls (n = 5 mice/genotype, representative example shown). (**C**) The rate of apoptosis in wildtype (WT) and Lx$^+$ transgenic thymocytes is unchanged in response to dexamethasone when plotted as the percentage of live cells over time. Live cell percentages were defined as negative for both AnnexinV and propidium iodide and quantified by flow cytometry. (**D**) *In vitro* survival of wildtype thymocytes (WT) and Lx$^+$ transgenic thymocytes in the absence of dexamethasone are also equivalent to one another.

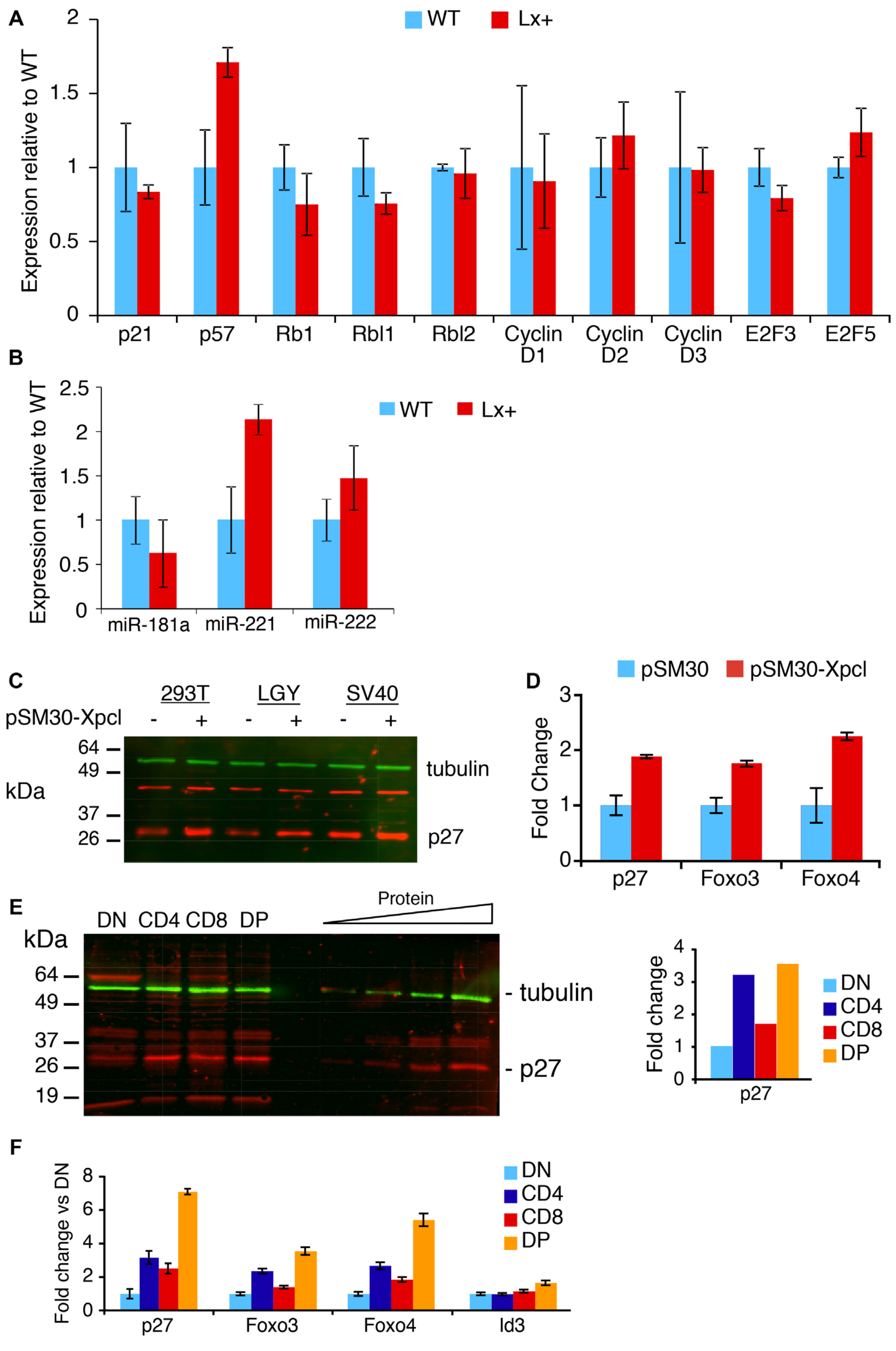

Supplementary Figure 4: (**A**) Expression of cell cycle genes in wildtype and Lx⁺ thymus ($n = 4$) quantified by SYBR green RT-qPCR. All of the tested genes, with the exception of Cyclin D1, are Targetscan (www.targetscan.org) predicted targets of the miR-106a~363 miRNA cluster. (**B**) Expression of miRNAs known to target $p27^{Kip1}$ in wildtype and Lx⁺ thymus ($n = 4$) quantified by SYBR green RT-qPCR. (**C**) Western blot of two murine T cell lines (LGY-6871 and SV40-180) transfected with Xpcl1 expression vector (+), vs. empty vector (−), demonstrates elevated p27 protein expression. (**D**) RT-qPCR shows increased RNA levels of p27, Foxo3, and Foxo4 in SV40-180 cells transfected with pSM30-Xpcl. (**E**) Wildtype thymocytes flow sorted by CD4/CD8 subsets and assayed by western for p27 protein expression. Differential expression of p27 protein in T cell subsets quantified normalized to tubulin loading control, and expressed relative to DN cells. A dilution series ("protein") of whole thymus confirmed assay linearity (**F**) Expression of $p27^{Kip1}$ mRNA measured by RT-qPCR in wildtype thymocyte subsets, expressed relative to DN T cells, mirrors that of Foxo3 and Foxo4.

Supplementary Table 1: miRNA hairpin RT primers and SYBR green qPCR primers

Hairpin RT Primers	Sequence
mmu-miR-106a, 20b, 17, 93, 20a*	GTCGTATCCAGTGCAGGGTCCGAGGTATTCGCACTGGATACGACTACCTG
mmu-miR-19b, 19a	GTCGTATCCAGTGCAGGGTCCGAGGTATTCGCACTGGATACGACTCAGTTTTGCAT
mmu-miR-92a	GTCGTATCCAGTGCAGGGTCCGAGGTATTCGCACTGGATACGACCAGGCC
mmu-miR-363	GTCGTATCCAGTGCAGGGTCCGAGGTATTCGCACTGGATACGACTTACAG
mmu-miR-106b	GTCGTATCCAGTGCAGGGTCCGAGGTATTCGCACTGGATACGACATCTGC
mmu-miR-25	GTCGTATCCAGTGCAGGGTCCGAGGTATTCGCACTGGATACGACTCAGAC
miRNA qPCR Primers	Sequence
mmu-miR-106a	CTGTGAAGAGGCAAAGTGCTAAC
mmu-miR-106b	CTGTCAAGTGGTAAAGTGCTGAC
mmu-miR-17	CTGTCAAGTGGCAAAGTGCTTAC
mmu-miR-20a	CTGTCAAGTGGTAAAGTGCTTAT
mmu-miR-20b	CTGTCAAGTGGCAAAGTGCTCAT
mmu-miR-19b	CGGCTGTGCAAATCCATGCAA
mmu-miR-19a	CGGCTGTGCAAATCTATGCAA
mmu-miR-92a	GTCTGGTATTGCACTTGTCC
mmu-miR-93	TGTCAAGTGGCAAAGTGCTGTTC
mmu-miR-25	GTCTGGCCATTGCACTTGTCT
mmu-miR-363	GTCTCATTGCACGGTATCCATCTGTA
Universal Reverse Primer	GTGCAGGGTCCGAGGT

*A single RT primer is compatible with multiple miRNAs.

Supplementary Table 2: Gene SYBR green qPCR primers

Name	Symbol	Primer F	Primer R
E2F3	E2f3	CGCGGTATGATACGTCCCTC	GCTGCCTTGTTCAGATCCAGG
E2F5	E2f5	CCTGAATGGCCAACAGTGTA	GTGGCTACAGCAAAGCATCA
p21	Cdkn1a	GACAAGAGGCCCAGTACTTCC	CAATCTGCGCTTGGAGTGATA
p27	Cdkn1b	AGGAGAGCCAGGATGTCAGCG	GCTGTTTACGTCTGGCGTCGA
p57	Cdkn1c	GTTCTCCTGCGCAGTTCTCT	GAGCTGAAGGACCAGCCTC
Rb	Rb1	ATAAGTCACGTAGGCGCACA	GATGCTCATCTGACCGGAGT
p107	Rbl1	GGAAAGTACGGGGTGAGCTA	AATGGTCCAGGAAACACGAC
p130	Rbl2	GTTGGAGTCTCTCCTGTCCG	TGAAGCAATGCCTTCTCCTC
Id3	Id3	GGAGAGAGGGTCCCAGAGTC	TCTCCAAGGACAAGAGGAGC
FoxO3a	Foxo3	CTGGGGGAACCTGTCCTATG	TCATTCTGAACGCGCATGAAG
FoxO4	Foxo4	CTTCCTCGACCAGACCTCG	ACAGGATCGGTTCGGAGTGT
Cyclin D1	Ccnd1	GCGTACCCTGACACCAATCTC	CTCCTCTTCGCACTTCTGCTC
Cyclin D2	Ccnd2	GAGTGGGAACTGGTAGTGTTG	CGCACAGAGCGATGAAGGT
Cyclin D3	Ccnd3	CGAGCCTCCTACTTCCAGTG	GGACAGGTAGCGATCCAGGT

Supplementary Table 3: Specificity of miRNA qPCR primers vs. target oligonucleotides

Primer vs Target	Fold difference in detection
106a vs 106b	7940.2
106a vs 17	135.9
106a vs 20a	∞
106a vs 20b	46.9
106b vs 106a	1622.7*
106b vs 17	9733.0*
106b vs 20a	7505.5*
106b vs 20b	∞*
17 vs 106a	2.6
17 vs 106b	227.6
17 vs 20a	188.4
17 vs 20b	753.6
20a vs 106a	∞
20a vs 106b	∞
20a vs 17	∞
20a vs 20b	∞
20b vs 106a	∞
20b vs 106b	∞
20b vs 17	∞
20b vs 20a	∞
19a vs 19b	134.4
19b vs 19a	18.4
92a vs 25	132.9*
92a vs 92b	2.6*
25 vs 92a	6194.1*
25 vs 92b	40563.3*

∞: No Amplification.
*RT Primer confers additional specificity.

Quantitation of miRNA and mRNA

Total RNA was isolated with Trizol (Invitrogen) according to manufacturers instructions, with 0.75 v/v of isopropanol, or by miRNeasy mini kit (Qiagen). Quantification of miRNA was performed using a stem-loop RT primer, followed by qPCR, as previously described (primers listed in Supplementary Table 1). mRNA was quantified by oligo-dT RT, followed by qPCR (primers listed in Supplementary Table 2). (Chen 2005) The specificity of each miRNA assay was confirmed (Supplementary Table 3) with ssDNA oligonucleotide standards matching the full length miRNA RT products. The specificity assays utilized 100 million copies of the oligonucleotide standards as template per miRNA qPCR reaction as previously described.(Chen 2005) Additional specificity conferred by the stem-loop RT primers was not quantified.
